# Screening and treatment of maternal genitourinary tract infections in early pregnancy to prevent preterm birth in rural Sylhet, Bangladesh: a cluster randomized trial

**DOI:** 10.1186/s12884-015-0724-8

**Published:** 2015-12-07

**Authors:** Anne CC Lee, Mohammad A. Quaiyum, Luke C. Mullany, Dipak K. Mitra, Alain Labrique, Parvez Ahmed, Jamal Uddin, Iftekhar Rafiqullah, Sushil DasGupta, Arif Mahmud, Emilia H. Koumans, Parul Christian, Samir Saha, Abdullah H. Baqui, for the Projahnmo Study Group

**Affiliations:** Department of Pediatric Newborn Medicine, Brigham and Women’s Hospital, 75 Francis Street, Boston, MA 02115 USA; Department of International Health, International Center for Maternal and Newborn Health; Johns Hopkins Bloomberg School of Public Health, 615 N Wolfe St, Baltimore, MD 21205 USA; International Centre for Diarrheal Diseases- Bangladesh, Centre for Reproductive Health, Mohakhali, Dhaka, 1212 Bangladesh; Department of Microbiology, Child Health Research Foundation, Dhaka Shishu Hospital, Sher-E-Banglanagar, Dhaka, 1207 Bangladesh; Center for Disease Control, 1600 Clifton Road, Atlanta, GA 30329 USA

**Keywords:** Urinary tract infection, Bacterial vaginosis, Abnormal vaginal flora, Preterm birth, Stillbirth, Miscarriage, Cluster randomized trial, Bangladesh

## Abstract

**Background:**

Approximately half of preterm births are attributable to maternal infections, which are commonly undetected and untreated in low-income settings. Our primary aim is to determine the impact of early pregnancy screening and treatment of maternal genitourinary tract infections on the incidence of preterm live birth in Sylhet, Bangladesh. We will also assess the effect on other adverse pregnancy outcomes, including preterm birth (stillbirth and live birth), late miscarriage, maternal morbidity, and early onset neonatal sepsis.

**Methods/Design:**

We are conducting a cluster randomized controlled trial that will enroll 10,000 pregnant women in Sylhet district in rural northeastern Bangladesh. Twenty-four clusters, each with ~4000 population (120 pregnant women/year) and served by a community health worker (CHW), are randomized to: 1) the control arm, which provides routine antenatal and postnatal home-based care, or 2) the intervention arm, which includes routine antenatal and postnatal home-based care plus screening and treatment of pregnant women between 13 and 19 weeks of gestation for abnormal vaginal flora (AVF) and urinary tract infection (UTI). CHWs conduct monthly pregnancy surveillance, make 2 antenatal and 4 postnatal home visits for all enrolled pregnant women and newborns, and refer mothers or newborns with symptoms of serious illness to the government sub-district hospital. In the intervention clusters, CHWs perform home-based screening of AVF and UTI. Self-collected vaginal swabs are plated on slides, which are Gram stained and Nugent scored. Women with AVF (Nugent score ≥4) are treated with oral clindamycin, rescreened and retreated, if needed, after 3 weeks. Urine culture is performed and UTI treated with nitrofurantoin. Repeat urine culture is performed after 1 week for test of cure. Gestational age is determined by maternal report of last menstrual period at study enrollment using prospectively completed study calendars, and in a subset by early (<20 week) ultrasound. CHWs prospectively collect data on all pregnancy outcomes, maternal and neonatal morbidity and mortality.

**Implications/Discussion:**

Findings will enhance our understanding of the burden of AVF and UTI in rural Bangladesh, the impact of a maternal screening-treatment program for genitourinary tract infections on perinatal health, and help formulate public health recommendations for infection screening in pregnancy in low-resource settings.

**Trial registration:**

The study was registered on ClinicalTrials.gov:NCT01572532 on December 15, 2011. The study was funded by NICHD: R01HD066156.

## Background

About 15 million babies are born preterm (born <37 weeks of gestation) annually [1], and preterm birth is the leading cause of neonatal and under-5 child mortality globally, accounting for one million neonatal deaths annually [2, 3]. Eleven million preterm births and the vast majority of deaths due to preterm birth complications occur in low-income countries, where there are limited resources and capacity for prevention and management. One third of preterm survivors suffer from severe long term neurological disabilities (e.g., cerebral palsy or mental retardation) [4, 5], and preterm infants carry increased risk of behavioral problems, school learning difficulties, chronic lung disease, retinopathy of prematurity, hearing impairment, and lower growth attainment [5]. Few interventions effectively prevent preterm birth [6], and the incidence of preterm birth is rising, in both low- and high-income countries [1, 7–11].

Treatment of maternal infections is a critical target for the prevention of preterm birth, particularly in low-income settings. Genitourinary tract infections may affect up to 41 % of women of reproductive age globally, and as many as 60–80 % of these infections in pregnancy are asymptomatic [12]. Maternal genitourinary tract infections have been significantly associated with a wide range of adverse perinatal and maternal outcomes, including miscarriage, stillbirth, preterm birth, fetal growth restriction, neonatal and puerperal sepsis, neonatal encephalopathy and neonatal and maternal mortality [13–16]. In developing countries where antenatal care coverage is limited [17], maternal infections are inadequately diagnosed and treated. Lower genital tract infections may ascend the reproductive tract and seed the amniotic cavity, which can trigger an inflammatory cascade eventually resulting in a number of adverse outcomes including preterm birth, chorioamnionitis, fetal growth restriction, stillbirth, puerperal sepsis and early onset sepsis. Maternal infection accounts for an estimated 50 % of preterm births [18], thus timely diagnosis and treatment of maternal infections is a prime target for the prevention of preterm birth, as well as other adverse pregnancy outcomes [19].

Abnormal vaginal flora (AVF), including bacterial vaginosis (BV), is the most prevalent vaginal infection in pregnancy, and is significantly associated with increased risk of preterm birth. AVF is triggered by an imbalance in the concentrations of endogenous vaginal microflora—a reduction in normal lactobacilli and the opportunistic overgrowth of *Gardnerella vaginalis* and other anaerobic organisms [20, 21]. Nugent et al. defined a scoring system (0–10) for vaginal flora based on a weighted combination of the relative concentrations of 3 bacterial morphotypes: *Lactobacillus*, *Gardnerella* or *Bacteroides*, and curved gram-variable rods (*Mobiluncus*) [22] [BV = Nugent score ≥ 7, intermediate flora: Nugent score 4–6, and AVF: Nugent score ≥4]. In a meta-analysis of 32 studies, asymptomatic BV was associated with a 6.32 times elevated risk of late miscarriage (95 % CI 3.65–10.94) and 2.16 times increased risk of preterm birth (95 % CI 1.56–3.00) [23]. Furthermore, intermediate vaginal flora is a heterogeneous condition which comprises ~15 % of all AVF [24], and has been associated with elevated risk of preterm birth and neonatal infections [25–28]. Among women with a prior history of preterm birth, Hauth and colleagues found that screening and treatment of asymptomatic BV with metronidazole and erythromycin at 22 weeks gestation significantly reduced the incidence of preterm birth from 46 to 31 % in the treatment group [29]. In the multi-center National Institute of Child Health & Human Development (NICHD) BV trial, 1953 average-risk women with asymptomatic BV between 16 and 24 weeks of gestation were randomized to receive two doses of metronidazole (2 g) or placebo. Treatment, however, did not significantly affect preterm delivery or other adverse perinatal outcomes [30]. In a more recent trial, Ugwumadu et al. reported that early (12–22 weeks of gestation) screening and treatment for AVF with 5 days of oral clindamycin resulted in a significant reduction in spontaneous preterm birth rate (12 % in placebo vs. 5 % in treatment group) and late miscarriage (13–24 weeks; 4 % in placebo vs. 1 % in treatment group) [24]. Lamont et al. randomized women with AVF between 13 and 20 weeks gestation to receive intravaginal clindamycin cream or placebo between 13 and 20 weeks gestation, and found that among infected women, there was a significant reduction in preterm birth incidence (10 % in control vs. 4 % in treatment group) [31]. Potential explanations for the treatment effect in the two latter trials may include: 1) the earlier timing of treatment, prior to the amniotic membranes sealing the uterus at 20 weeks [32], which may prevent early ascension of bacteria into the intrauterine cavity; 2) antibiotic choice: 5–7 day course of clindamycin, which has greater activity against *Mobiluncus* and atypical *Mycoplasma* species vs. 2 days of metronidazole [33]; and 3) treatment of AVF in Ugwumadu et al. and Lamont et al. vs. treatment of BV only in the average-risk NICHD trial. A Cochrane meta-analysis including 15 trials (n=5888 women) concluded that BV treatment across all gestational ages did not significantly reduce preterm birth, largely due to heterogeneity in definition, timing of treatment and choice of antibiotic [33]. However, the risk of preterm birth was significantly reduced by treatment of BV at <20 weeks gestation (5 trials in 2387 women; OR 0.63, 95 % CI 0.48–0.84) and treatment of AVF (2 trials, 894 women; OR 0.51, 95 % CI 0.32–0.81). Thus, in low-resource settings such as rural Bangladesh, where BV and preterm birth are prevalent, treatment of AVF in early pregnancy may hold promise in reducing the incidence of preterm birth. Evaluation in a well-conducted population-based randomized trial is needed.

Urinary tract infection (UTI) is also prevalent in pregnancy and associated with preterm birth. Uropathogens tend to be gram-negative bacteria with virulence factors and endotoxins, which may trigger the inflammatory cascade and preterm delivery. The prevalence of asymptomatic bactiuria in pregnancy may range from 7 % to as high as 86.6 % [34, 35]. Approximately 30 % of women with untreated bactiuria develop pyelonephritis [36], and before the era of antibiotics, 30–50 % of women with pyelonephritis delivered preterm [37]. In an early meta-analysis by Romero et al., asymptomatic bactiuria carried a 2-fold elevated risk of preterm delivery (95 % CI 1.43–2.77) [38]. In the Cardiff Birth Survey, asymptomatic bactiuria was not associated with all preterm births (OR 1.2, 95 % CI 0.9–1.5); however there was a significant association with medically indicated preterm birth (OR 2.0; 95 % CI 1.5–2.8) [39]. In a meta-analysis of randomized clinical trials for asymptomatic bactiuria, antibiotic treatment reduced the risk of low birth weight (RR 0.56, 95 % CI 0.43–0.73; *n* = 8 studies) [38], however there are inadequate data on the effect on preterm birth.

## Methods/Design

### Trial design and preparation

#### Aim

The ***primary aim*** of this study is to determine the impact of community-based screening and treatment of AVF and UTI in early pregnancy (13–19 weeks) on the incidence of preterm live birth in Sylhet district, Bangladesh.

The ***secondary aims*** of this study are:

- To determine the population-based impact of community-based screening and treatment of AVF and UTI on the:
  - proportion of pregnancies with outcomes occurring prior to 37 weeks including late miscarriage (pregnancy losses 20 to <28 weeks) and preterm birth (stillbirth and live birth 28 to  <37 weeks);
  - proportion of babies with early onset neonatal sepsis
  - proportion of pregnancies with maternal morbidity (clinical infections including pyelonephritis, puerperal sepsis)
  - proportion of babies born with low birth weight or small for gestational age
  - neonatal mortality rate
- To determine the prevalence of AVF and UTI, including asymptomatic bactiuria, among pregnant women in Sylhet district, Bangladesh.
- To evaluate the accuracy of simple, low-cost, point of care diagnostic tests for detecting BV and UTI by community health workers (CHWs) in a rural, developing country setting.

### Study site and population

**Fig. 1:**
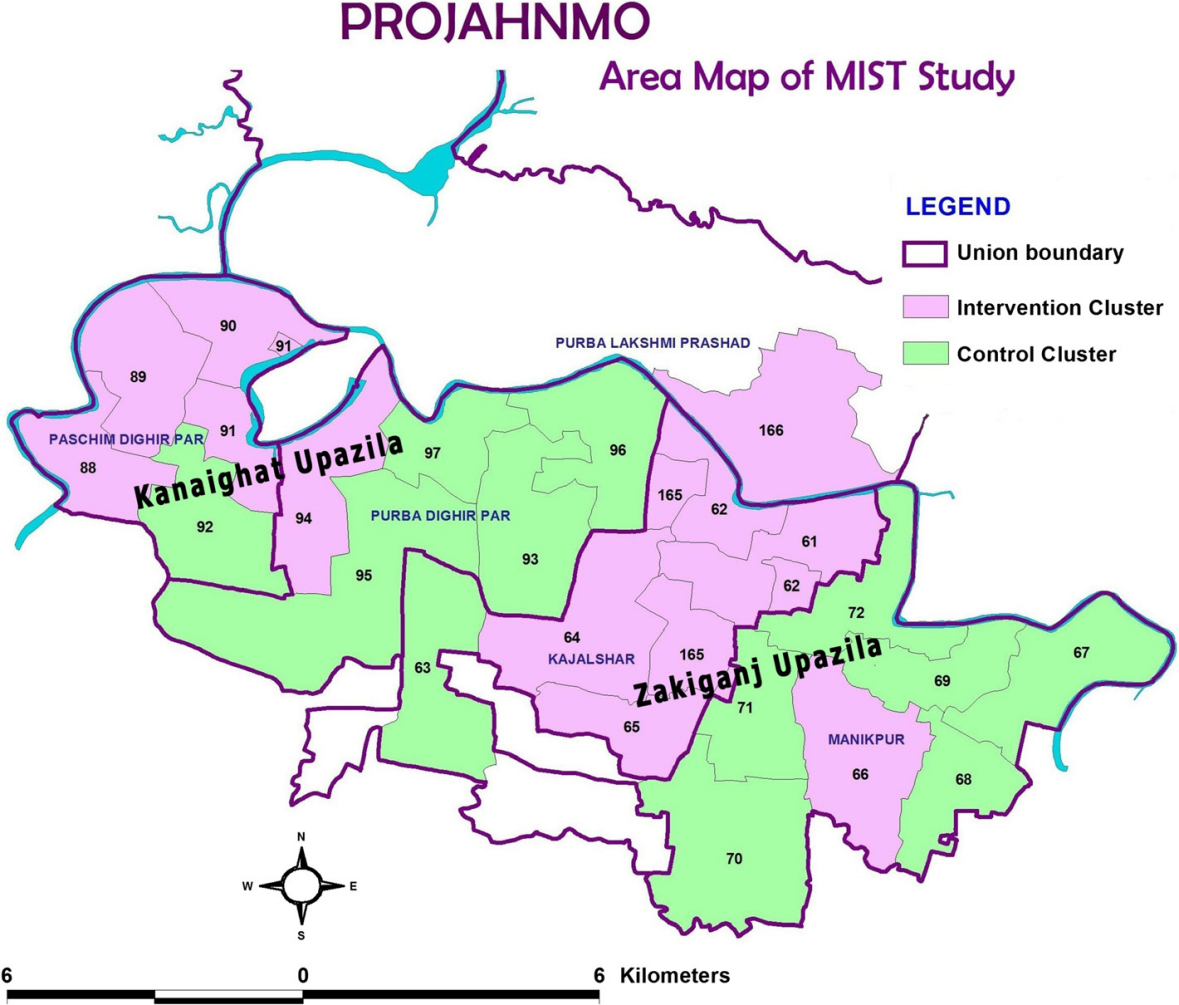
Map of Sylhet District, Bangladesh and unions participating in the Projahnmo MIST trial

The Maternal Infection Screening and Treatment (MIST) study is being conducted in the Projahnmo research site in two sub-districts (*upazillas*; Zakiganj and Khanaighat) of Sylhet District in Bangladesh (Fig. 1, Map). Projahnmo is a collaboration of the Ministry of Health and Family Welfare (MOHFW) of the Government of Bangladesh, the International Centre for Diarrhoeal Disease Research, Bangladesh (iccdr,b), Shimantik (a non-governmental organization), Child Health Research Foundation, Brigham and Women’s Hospital (Harvard Medical School), and the Johns Hopkins Bloomberg School of Public Health. The estimated population of the MIST study area is about 100,000. The burden of prematurity, neonatal sepsis, neonatal death, and stillbirth is high in this population (preterm birth rate: 22 %, clinical neonatal sepsis: 9.9 %, neonatal mortality rate: 28/1000 live births, stillbirth rate: 38/1000 births) [40, 41]. Health services in Bangladesh are provided by the MOHFW, NGOs and private providers. In the government sector, two community-based workers, a family welfare assistant and a health assistant together serve a population of 6000–7000. First level outpatient clinics, called Health and Family Welfare Centres, serve a population of about 25,000, with one clinic per union (lowest local government entity in Bangladesh). Sub-district hospitals with both inpatient and outpatient facilities serve a population of about 250,000.

The study area is served by 24 project CHWs, who are women with at least a 10th grade education, are residents of the study community, and have received 6 weeks of training on basic maternal and newborn care. Within each CHW cluster area, there are additionally Village Health Workers (VHWs), one in each village with a population of ~1000. Each cluster area is assigned to 1 CHW and 4–5 VHWs to serve an estimated 120 pregnancies and 116 live births annually; this is feasible because the trial area has very high population density, and the same ratio of population to CHWs/VHWs has been used in our previous and current studies in Sylhet.

We conducted a complete census and socioeconomic survey of the study population in 2009 and continue to conduct health and demographic surveillance every two months. All households and women of childbearing age have unique current and permanent identification numbers, which allows individual tracking and longitudinal follow-up.

### Pregnancy surveillance, eligibility, and enrollment (Fig. 2)

**Fig. 2:**
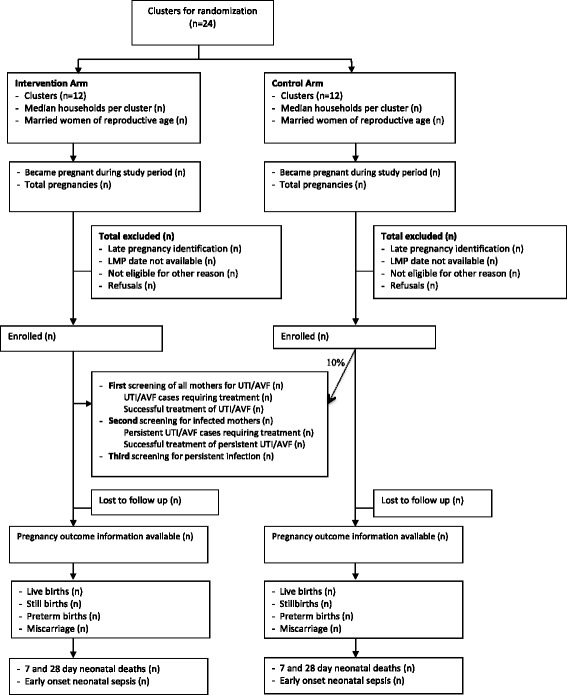
Trial design flowchart

All women who become pregnant in the study area are potentially eligible to participate in the trial. Prior to the start of the study, all women of reproductive age (15–49 yo) in the study area were provided study calendars with instructions and training by CHWs to prospectively circle the first day of each menstrual cycle. VHWs conduct monthly pregnancy surveillance visits where mothers are asked if their last menstrual period (LMP) was greater than 4 weeks ago. If LMP was >4 weeks, a CHW conducts a home visit to perform a urine pregnancy test. Enrollment may begin as soon as 5 weeks gestation and continues until 19 weeks gestation. At the time a missed period is detected during screening for study eligibility. Subjects are excluded from the study if they have no or uncertain recall of LMP (due to lactational amenorrhea, recent discontinuation of contraceptive or irregular menses), LMP >19 weeks, history of irregular bleeding due to injectable Depo-Provera, or self-reported history of severe chronic disease based on a medical history checklist. Participants are enrolled after final eligibility is confirmed by the CHW and oral consent is obtained.

### Basic antenatal-postpartum care package

In all study areas, CHWs provide basic antenatal and postpartum care. This consists of two antenatal home visits (13–19 weeks and 28–32 weeks of gestation) and four postnatal home visits (1st, 3rd, 7th, and 28th day of life) to all enrolled pregnant women. During antenatal home visits, CHWs provide: counseling on birth and newborn care preparedness (importance of antenatal check up, use of a skilled/trained birth attendant, essential newborn care, danger signs, care seeking, birth spacing), demonstrations (hand washing, cord cutting, immediate newborn care), facilitation (emergency savings, selection of birth attendant and newborn care person), supply (clean delivery kit, iron folate tablets) and referral (for routine antenatal care, tetanus toxoid immunization, maternal danger signs). Postnatal visit components include: counseling (maternal and newborn danger signs, essential newborn care, and exclusive breast feeding), assessment of the newborn (morbidity and breastfeeding), referral (for newborn and maternal danger signs, postpartum contraceptive methods), demonstration/support (breastfeeding techniques) and supply (contraceptives).

### Randomization

The units of randomization in this trial are the 24 CHW areas, each comprising several adjacent villages. Allocation to the intervention vs. control arms was done via a restricted randomization procedure. All $$ \left(\begin{array}{c}\hfill 24\hfill \\ {}\hfill 12\hfill \end{array}\right) $$ possible randomization sequences were generated where each sequence allocated 12 of the CHW areas to either intervention or control. Using data on births and preterm status from our prior study in this area [42], we restricted the set of eligible sequences to those where intervention vs. control ratios of predicted preterm birth and predicted total births were within 0.975 to 1.025. An additional criterion for retaining a sequence as eligible was that the 13 CHW areas that had participated in a prior study on birth spacing (“The Health Fertility Study”) were allocated equally to intervention and control (i.e. 6 and 7 or 7 and 6). Among the 2,704,156 sequences, 164 sequences met the criteria, from which a single sequence was randomly selected. Workers and participants in the study are not blinded to the intervention group but are not aware of the study aims/hypotheses.

### Screening and treatment intervention

The screening and treatment intervention is provided to all pregnant women enrolled in the intervention clusters. Furthermore, to examine the comparability of baseline infection prevalence between intervention and control areas, a random 10 % of mothers enrolled in the control arm are selected to receive the screening and treatment intervention. To select the 10 % of mothers from control areas, each CHW initially randomly selected a number between 1 and 10 which indicated the starting number of the women in her area to receive the intervention. From that starting point every 10th enrolled mother was included in the group to receive the screening and treatment intervention. The sample size was not adjusted for this allocation.

### Screening

CHWs collect samples of vaginal flora and urine during home visits between 13 and 19 weeks gestation. Vaginal specimens are collected via sterile self-administered vaginal swabs. Self-administered vaginal swabs have been used with high acceptability and quality in diverse populations including in Bangladesh, providing cost-savings and cost effectiveness for STI testing [43–46]. Women are instructed by the CHW to insert a Dacron swab ~4–5 cm into the vagina, allow the swab to stand for 15 s, and rub the lateral walls of the vagina for 4–5 s prior to withdrawal [47]. The CHW gently rolls the swab onto a plain glass slide and allows it to air dry prior to transport to the Sylhet field laboratory. A clean catch midstream urine specimen is obtained for urine culture. The CHW instructs the mother to spread the labia widely before collecting 20–30 mL of the midstream urine into a sterile wide-mouthed container.

### Specimen storage and transport

All specimens collected in the field by CHWs are immediately labeled and stored in a cooler refrigerated with ice packs (~2–8 °C) and transported to the Sylhet field laboratory. In the field laboratory, the vaginal smears are Gram stained within 1–2 days of plating [22]. Urine specimens are inoculated on sheep blood agar plates in the Sylhet field laboratory within 6 h of collection for incubation.

### Diagnosis of abnormal vaginal flora

Abnormal vaginal flora are classified by microscopic examination of a Gram stained sample of the vaginal smear and Nugent scored (Table 1) [22]. Nugent scores between 7 and 10 are classified as BV, and scores between 4 and 6 are classified as intermediate flora; all scores ≥ 4 are classified as AVF [48]. The time window from specimen collection to Nugent scoring is 4 days, and from Nugent scoring to first dose of treatment is 2 days, for a total of 6 days from sample collection to treatment. Treatment is based on a single reading by a trained and standardized microbiologist. The laboratory staff are trained and standardized in Nugent scoring on a non-study sample of slides (*n* = 250) until there is high concordance of readings (kappa >0.8) between laboratory staff and gold standard scorers, and high sensitivity and specificity of AVF and BV compared to the gold standard reader (>85 %) prior to the initiation of the study.

**Table 1:** Nugent scoring for vaginal flora

*Lactobacillus* Morphotypes^a^	*Gardnerella* / *Bacteroides* Morphotypes^a^	Curved gram-variable rods
Score 0 for >30	Score 0 for 0	Score 0 for 0
Score 1 for 15–30	Score 1 for <1	Score 1 for <5
Score 2 for 14	Score 2 for 1–4	Score 2 for 5+
Score 3 for <1	Score 3 for 5–30	
Score 4 for 0	Score 4 for >30	

^**a**^Average count per high powered field (1000× oil immersion), viewing at least 10–20 fields

### Diagnosis of urinary tract infection

Urine specimens are plated on standard MacConky and Blood agar plates and incubated for 48 h. Bacterial growth is speciated using standard microbial techniques, and antibiotic sensitivity patterns are determined. Bacterial growth is defined in the following categories: 1) ***high-burden urinary tract infection***: bactiuria of >10^5^ colony forming units (CFU) per 1 mL of urine of a single uropathogen [49], 2) ***intermediate growth***: bactiuria with >10^3^–10^5^ CFU/mL of a single uropathogen, and 3) ***contamination***: bacterial growth of >1 organism OR growth of a non-urinary tract pathogen. At the time of the specimen collection visit, CHWs inquire about maternal symptoms of UTI (dysuria, urinary frequency, hematuria, abdominal pain, fever, flank pain). ***Symptomatic intermediate growth*** is defined as mothers with intermediate burden growth and UTI symptoms. ***Cystitis*** is diagnosed in women with positive urine culture (high burden or intermediate growth) and symptoms of dysuria, urinary frequency, hematuria, urinary urgency or suprapubic tenderness, without upper urinary tract symptoms (fever, chills, flank or back pain) [49]. ***Pyelonephritis*** is diagnosed in women with positive urine culture and systemic symptoms (fever, chills, flank pain or back pain) [49].

### Treatment of abnormal vaginal flora and urinary tract infections

**Fig. 3:**
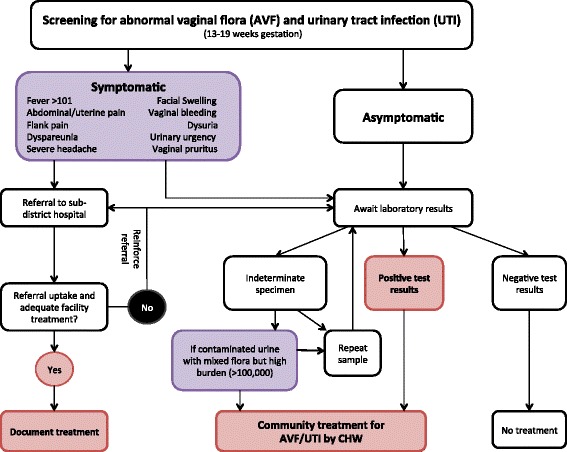
Referral and treatment algorithm for intervention clusters

Women who are clinically symptomatic at any antenatal or postnatal visit are referred to the sub-district hospital for full evaluation and treatment (Fig. 3). Women with symptoms of illness are visited on the following day to follow her clinical status and ensure referral compliance. CHWs conduct a home visit to women with positive test results within 48 h of receiving the results to initiate treatment. AVF is treated with oral clindamycin 300 mg per oral (po) twice daily (bid) for 5 days per the regimen used by Ugwumadu [24]. After the initial treatment course, women are rescreened 3 weeks after the first treatment. If the second vaginal specimen has Nugent score ≥4, the women are retreated with a second course of clindamycin 300 mg po bid for 5 days. For retreated women, a final vaginal sample is collected 3 weeks after the second course of antibiotics to document response to treatment.

**Fig. 4:**
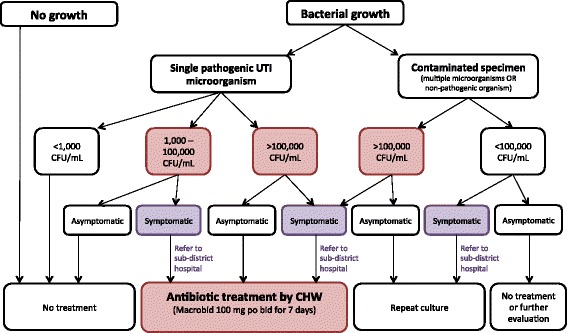
Urine culture treatment algorithm

The algorithm for treatment of positive urine cultures is shown in Fig. 4. All mothers with high burden UTI and symptomatic intermediate growth are treated. The initial antibiotic treatment is Macrobid/Nitrofurantoin 100 mg po bid for 7 days. Women diagnosed with pyelonephritis are referred to the sub-district hospital for further evaluation and management. For all positive urine cultures, a repeat urine culture is obtained 1 week after completion of antibiotics for test of cure. If the second urine culture is positive, the supervising field physician selects an appropriate antibiotic based on the prior culture’s antimicrobial sensitivity pattern. Persistent UTIs are referred to Sylhet Osmani Medical College Hospital for evaluation and management.

### Point of care testing validation

On a subsample of women (*n* = 1386), point of care tests are conducted in the field site at the same time as the first specimen collection. An additional vaginal swab is taken for rapid testing with BVBLUE (Rapid Sialidase test, Genzyme, Cambridge, MA). An additional 2 mL aliquot of urine is tested with the URISCREEN test (rapid catalase screen, URISCREEN, Jant Pharmacal, Encino, CA).

### Medication adherence and monitoring

The VHWs and CHWs monitor all women who are prescribed medication for medication adherence and adverse events. The CHW observes the mother take the first antibiotic dose and provides her with a medication punch card to indicate every dose taken. The CHW visits the mother 2–3 days after starting the antibiotic to screen for symptoms of *C. difficile* diarrhea/colitis (associated with clindamycin) and hemolytic anemia (associated with Macrobid). At the end of the antibiotic course, the CHW collects the medication punch card, conducts a pill count, and assesses compliance and response to therapy based on the mother’s history.

### Post-natal visits

CHWs in both intervention and control clusters are notified of all births through a community-based notification system which relies on families and VHWs. During the first postnatal visit, the CHW records the date and outcome of delivery, including stillbirths (pregnancy losses ≥28 weeks) or late miscarriages (pregnancy losses 20 to <28 weeks). If a stillbirth has occurred, the CHW notifies a supervisory staff member who returns to conduct a verbal autopsy module for stillbirth [50]. For all births, CHWs record the vital status of mother and baby, and basic information regarding characteristics of labor and delivery, including data on timing and duration of labor, timing (before or after onset of labor) and length of rupture of membranes, maternal fever, and other morbidity before, during, and after delivery. For each live newborn delivered, the newborn's sex, weight, and care given during and immediately after birth (bathing, massage, cord care, breastfeeding practices) are recorded.

At the first postnatal visit, which occurs in most cases on the first day of life, CHWs weigh the infant with a digital weighing scale. At the start of the study, the KL-218 digital scale (precision 10 gm, Donghuan Electronic Manufacturing, Donghuan, China) was used. The scale was transitioned to the Tanita BD 585 digital pediatric scale in September 2015 (precision 10 gm, Tanita corporation, Toyota, Japan). Scales are calibrated daily prior to home visits. CHWs also measure head circumference, mid-upper arm circumference, infant length, and chest circumference. These measurements are repeated thrice (median value used for analysis). The general health status of the mother and infant are assessed by the CHW during postnatal visits. The mothers are assessed for vital status, fever, uterine tenderness and symptoms of postpartum hemorrhage. Women with symptoms of postpartum complications (fever >38.3 °C, abdominal/uterine tenderness, self-report of excessive hemorrhage) are referred to government sub-district hospitals for further management. Evaluation of the neonate is conducted according to an IMCI-type algorithm [51], and the CHW assesses the infant for temperature, respiratory rate, and signs and symptoms of severe illness. The CHWs conduct follow up postnatal visits at homes in which severe illness is detected in the mother or neonate within the next 24 h to monitor the mother/infant for signs of illness and reinforce the referral. If the ill mother/newborn has not sought hospital care, the CHW facilitates referral by helping to arrange transportation and payment of hospital fees. The CHW returns on days 3, 7, and 28 after birth to reassess and inquire about signs of morbidity in the mother and infant.

### Outcome measures

The primary and secondary study outcomes and definitions are shown in Table 2.

**Table 2:** Study outcomes for MIST trial

	Numerator	Denominator
Primary outcome		
Preterm live birth	Live births <37 weeks of gestation	All live births
Secondary outcomes		
Preterm birth and late miscarriage	Live births (28 to <37 weeks)	All birth outcomes ≥ 20 weeks
	Stillbirth (28 to <37 weeks)	
	Late Miscarriage (20 to <28 weeks)	
Late miscarriage rate	All spontaneous abortion/miscarriage (non-therapeutic abortions) occurring between 20 to <28 weeks	All birth outcomes ≥20 weeks
Stillbirth rate	Stillbirth (≥28 weeks) and no signs of life	All live births and stillbirths ≥28 weeks
Perinatal mortality rate	Stillbirth + early neonatal death (birth to 7 days)	All live births and stillbirths ≥28 weeks
Neonatal mortality rate	Neonatal death (birth to 28 days)	Live births
Early onset sepsis	Very severe disease by current IMCI guidelines on any day in first week of life	Live births

#### Gestational age

The primary measure of gestational age is determined by maternal report of the first day of her LMP, reported during monthly pregnancy surveillance and with the aid of a prospectively completed menstrual calendar. An early ultrasound assessment (<20 weeks gestation) was originally planned for a random sample of 20 % of the enrolled women. In year 3 (2013), we began to conduct ultrasonography on as many enrolled pregnancies as possible.

#### Preterm live birth

Preterm live birth is defined as a baby born alive <37 weeks gestation defined by LMP. Early preterm live birth is defined as a baby born alive <34 weeks gestation defined by LMP. The denominator for preterm live birth rates is all live births.

#### Pregnancies with outcomes occurring prior to 37 weeks

This measure is defined as the spontaneous termination of pregnancy from 20 to <37 weeks resulting in: 1) preterm live birth, 2) late miscarriage (spontaneous pregnancy loss 20 to <28 weeks) that is not due to induced abortion, or 3) preterm stillbirth (28 to <37 weeks). A preterm stillbirth is defined as an infant born without signs of life (no spontaneous crying, breathing, and/or movement) at 28 to <37 weeks gestation. The denominator for this outcome is all births ≥20 weeks.

#### Clinically suspected early onset neonatal sepsis

Early onset neonatal sepsis is defined using the neonatal IMCI algorithm for very severe disease [52] with onset in the first week of life.

#### Small for gestational age

Small for gestational age is defined as birth weight <10 % (measured within the first 72 h of life) for gestational age using the INTERGROWTH-21 newborn birthweight standard [53].

#### Low birth weight

Low birth weight is defined as birth weight (measured within the first 72 h of life) of <2500 g. We will also assess the outcome of birth weight <2000 g.

#### Maternal morbidity

Maternal morbidity outcomes are assessed primarily by self-report. We follow maternal history for clinical signs indicating probable pyelonephritis and endometritis.

#### Neonatal mortality

In the event of death of a newborn during the neonatal period, the CHW refers the case to a supervisory staff member who conducts an in-depth verbal autopsy using a revised version of the WHO standard verbal autopsy instrument which has been used extensively by our study team and CHWs at the study site [54–56]. Verbal autopsy data are reviewed by two independent physicians, and a consensus on proximate and underlying cause(s) is reached. Data are further analyzed for cause-specific mortality using a computer-based expert algorithmic approach [57, 58].

### Sample size and analysis

In order to calculate the sample size, we first hypothesized that a population-level reduction in the preterm rate in the range of 15–20 % would be of public health importance, given the importance of the outcome and the level of complexity of any future scaled- up programmatic implementation of the intervention. Once we established this desired effect size range, we worked through a series of calculations (with corresponding assumptions) to determine the number of babies required to achieve sufficient power (i.e. 80 %) for a population-level reduction in this range (i.e. 15–20 %).

Given that the intervention is directed only at a subset of the population, we first estimated the proportion of preterm birth among both infected and non-infected women. Estimation of these proportions requires three parameters:

1. The proportion of preterm in the population: we estimated this to be 20 %, based on our prior data from this population [51].
2. The proportion of women in the intervention clusters that actually receive treatment under the universal screening program, or equivalently an estimate of the total proportion of women with UTI and/or AVF (Nugent ≥4).
3. The ratio of preterm birth risk among infected and non-infected women; we estimated this ratio to be 2.5 from the literature.

From previous data and the literature, we estimated that about 15 % of women in this setting have either AVF, UTI, or both. Among those with AVF, some will fall into the intermediate flora score range (Nugent score 4–6) and some into the BV scoring range (Nugent score 7–10). Our best estimates of the relative proportions in these two subgroups was approximately 85 % within the BV (Nugent 7–10) range, with the remaining 15 % of women with AVF falling within the intermediate flora scoring range (Nugent 4–6) [24]. We based our prevalence estimates and treatment effect size on prior studies of similar screening and treatment programs for **all abnormal vaginal flora** (Nugent scores ≥4).

The last step in fixing our parameters and assumptions for sample size calculation was to examine the range of impacts that the intervention would need to afford ***among infected women*** in order to result in population-level impacts of 15 to 20 %. For a 50 % impact among infected women, we estimated a 15 % population-level reduction. For a 65 % impact on infected women, we estimated a 20 % population-level reduction. These impacts are consistent with those from prior studies that have demonstrated the impact of treatment of women with AVF (Nugent ≥4) is an approximately 60 % reduction in preterm births (Lamont et al.: 60 %, Ugwamadu et al.: 58 %). Assuming this impact (i.e. 60 %) among infected women to be similar to the impact in this population, the population-level effect size would be 18.4 %.

Having selected an effect size of 18.4 % (i.e. relative reduction in the population rate) we proceeded to estimate the sample size required. As the number of clusters available is fixed (*n* = 24), we followed an approach where first the sample size required under a naïve assumption of individual randomization was calculated, and then inflated by a factor (“design effect”) in order to account for correlation within CHW areas arising from the cluster-randomized design. We estimated this design effect as (1+ ***ρ*** •[ ***θ*** -1+ ***θ γ***^2^]), where ***ρ*** is the estimated intra-cluster correlation coefficient, ***θ*** is the mean sample size per cluster under individual randomization, and ***γ*** is the coefficient of variation in the cluster size [59]. Among ~16,000 births between June 2007 and September 2008 in this trial area, the intra-cluster correlation coefficient in this setting was 0.0060 (95 % CI: 0.0025–0.0095) and the coefficient of variation in cluster size was 0.33, leading to a design effect of 1.95 for preterm live birth. Taking this design effect into account, the number of live births required in each group to observe the estimated 18.4 % relative reduction with 80 % power and allowing 5 % Type I error is approximately 3367. Finally, we assumed that 90 % of the women screened at 13–19 weeks would have a live birth and 10 % would be lost to follow up, requiring enrollment of a total of 8314 in both control and intervention areas at the 13–19 week visit. Given a total population size of ~100,000 and a crude birth rate of 27/1000, we project that this sample will be reached in approximately 37 months, with total field work and follow-up of about 4 years.

### Data management

Data are collected on paper forms by CHWs and other field workers during home visits with enrolled mothers and their newborn babies. Forms are checked for accuracy and completeness by field supervisors prior to transport for data entry in Dhaka. A data entry prioritization protocol enables time-sensitive data, such as results from screening tests or treatment follow up data, to be entered locally at the field site/laboratory; this enables minimal turn-around time between screening and treatment, and allows real-time assessment of compliance and monitoring of treatment-related adverse events. All forms are eventually entered into a secure Oracle database using customized data entry screens, with built-in range and validation checks. The database is backed up daily and a de-identified version is transferred (using encrypted peer-to-peer transfer software) on a regular basis to Baltimore, USA for archiving and creation of merged analytic files.

### Analysis

Interim (i.e. for DSMB analyses) and final analyses will follow the same protocol. First, we will present descriptive information on the recruitment, enrollment, and follow up of women and their newborns (i.e. a participant flowchart). We will then assess characteristics of the enrolled pregnancies to determine the extent to which our randomization procedure achieved comparability; these characteristics will include maternal, paternal, household, and socio-economic variables. For clusters allocated to the intervention arm we will describe the coverage of the screening and treatment intervention, including the distribution of time (i.e. gestational age) at screening, results of the initial and follow up vaginal swab (overall AVF, and intermediate vs. bacterial vaginosis) and urine tests (normal, intermediate asymptomatic, intermediate symptomatic, and high-burden single growth), and compliance with treatment. For the 10 % sub-sample of women in the control clusters who are randomly selected for the screening and treatment intervention, we will generate a similar set of intervention/coverage related indicators and compare these with those of the larger group of women in the intervention clusters.

The next stage of analysis will focus on describing the impact of the intervention on the primary and secondary outcomes. The gestational age at birth will be defined for each pregnancy as the number of days between last menstrual period (reported and recorded at enrollment) and the date of the pregnancy outcome. The primary study endpoint is preterm live birth, and thus we will present, in each group, the number of live births born prior to the pregnancy reaching 37 complete weeks (numerator) and the total number of live births (denominator). The preterm “rate” will be estimated as this proportion with a 95 % binomial exact confidence interval. The impact of the intervention will be assessed by estimating the relative risk of preterm live birth and the absolute risk difference using binomial regression models with a log and identity link functions, respectively. The standard error of this estimate will be constructed using generalized estimating equations and a 95 % confidence interval calculated. We will follow a similar analytic approach to estimate live births prior to 34 weeks (by group and with relative risk) and to estimate the average gestational age at birth for live births, for which we will use a linear regression model with an identity link function and GEE to estimate errors.

We will then expand the pool of analyzable pregnancies to include all spontaneous delivery (i.e. late miscarriages and stillbirths, in addition to live births) and follow an identical approach to estimate the impact of the intervention on this broader set of pregnancies. We will estimate stillbirth and neonatal mortality rates (very early: <1 day, early: <7 days, and overall neonatal mortality: <28 days), and compare across the groups using binomial regression models. Other secondary outcomes of interest that will be compared across the groups include early-onset neonatal infection (defined through use of a sign-based algorithm and restricted to the first 7 days of life) and maternal report of infection in the first 7 and 28 days after delivery.

These models will be presented without adjustment, and a second set of analyses will be conducted with adjustment for any variables found to be imbalanced between the groups (if necessary). Planned sub-analyses to examine for effect modification include stratifications by age at delivery and parity. All estimates of the intervention's impact will be presented with confidence intervals constructed from standard error estimates adjusted for the cluster randomization using GEE.

Additional planned analyses that are not related directly to the impact of the intervention include descriptive analyses of the organisms isolated from urine specimens, antibiotic resistance patterns, contamination rates over time, and adverse event reporting.

### Data safety and monitoring board and interim analysis

An independent Data Safety Monitoring Board (DSMB) consisting of a clinical trialist and infectious disease specialist, an obstetrician-gynecologist and a biostatistician is established for this trial. The DSMB met prior to the start of data collection to review and approve the study protocol, and met in person to review interim analysis after 33 % and 66 % of the study outcomes were achieved. At both meetings (February 2014, February 2015), the DSMB indicated the study should continue as planned. The DSMB reviewed the data collected in the study for evidence of issues related to safety of study subjects and the efficacy of the study interventions. The DSMB may also request further meetings, either face-to-face or by teleconference. At all meetings, minutes are kept of the deliberations of the DSMB and provided to the JHU Insitutional Review Board (IRB), the Ethical Review Committee of the International Centre for Diarrhoeal Diseases Research, Bangladesh, and Brigham and Women’s Hospital.

### Approvals

The study protocol was approved by the IRB of the Johns Hopkins Bloomberg School of Public Health, the Ethical Review Committee of the International Centre for Diarrhoeal Diseases Research, Bangladesh, and the IRB of Partners Health Care (Brigham and Women’s/Faulkner Hospital and Massachusetts General Hospital).

## Discussion

The Projahnmo MIST Study, expected to be completed at the end of 2015, is a cluster randomized trial that will evaluate the impact of an early pregnancy screening and treatment program for AVF and UTI on population-level rates of preterm birth, compared to a basic package of routine antenatal and postpartum care alone. Furthermore, we will evaluate the impact of the intervention on rates of neonatal and maternal postpartum infection, and determine the population-based prevalence of these important genitourinary tract infections in pregnancy in a rural low-income country setting in South Asia. Finally, we will determine the accuracy and potential utility of point-of-care diagnostics to diagnose these infections.

Maternal genitourinary tract infections are prevalent and substantially contribute to global maternal and neonatal morbidity and mortality, yet they are poorly quantified, detected, and treated in low-income settings. Few interventions have proven effective in the prevention of preterm birth at the population level in high-income settings. However, in low-resource settings, early detection and treatment of maternal genitourinary tract infections may hold promise as a potentially low-cost, high-impact intervention to prevent preterm birth. BV and UTI are the most common infections in pregnancy, and there is strong evidence of their association with preterm birth, low birth weight, and early onset neonatal sepsis [60, 61]. While treatment of asymptomatic AVF in pregnancy is not standard of care, two promising trials in the UK have shown that treatment of asymptomatic AVF may reduce preterm birth rates by up to 60 %. Treatment of asymptomatic bactiuria is considered standard of care in high-income settings, and may reduce low birth weight and maternal pyelonephritis. There are few studies assessing the impact of a community-based screening and treatment program for genitourinary tract infections in low-income settings, where the burden of disease and impact may arguably be the greatest [62]. The strengths of the proposed study are as follows: 1) this will be one of the first studies of the prevalence of AVF and UTI (including asymptomatic bactiuria) in a large, rural, community-based cohort of pregnant women in a developing country; 2) using a cluster randomized controlled design, we will determine the effect of treatment of AVF and UTI on preterm birth, because present data are heterogeneous and limited by study design; 3) we will utilize an established network of CHWs for the screening and treatment program, which may be a feasible and low-cost approach to delivering antenatal health services in similar developing country settings.

If the intervention is shown to be efficacious, the data from this trial will be used to contribute to the design of global public health strategies and recommendations regarding routine antenatal screening and treatment of maternal infections in low- and middle-income countries, and further our understanding of novel pathways to prevent preterm birth and reduce newborn morbidity and mortality in the highest burden settings.

## Abbreviations

AVF: Abnormal vaginal flora (Nugent Score 4–10)
BV: Bacterial vaginosis (Nugent Score 7–10)
IF: Intermediate flora (Nugent Score 4–6)
UTI: Urinary tract infection
TOC: Test of cure
BNCP: Basic antenatal-postpartum care package
CHW: Community health worker
POC: Point of care
